# Pre-exposure prophylaxis (PrEP) awareness, attitudes and uptake willingness among young people: gender differences and associated factors in two South African districts

**DOI:** 10.1080/16549716.2021.1886455

**Published:** 2021-02-19

**Authors:** Simukai Shamu, Patience Shamu, Sikhulile Khupakonke, Thato Farirai, Thato Chidarikire, Geoffrey Guloba, Nkhensani Nkhwashu

**Affiliations:** a Health Systems Strengthening Cluster , Foundation for Professional Development, Pretoria, South Africa; b School of Public Health, University of the Witwatersrand, Johannesburg, South Africa; c Wits Health Research Consortium, University of the Witwatersrand, Johannesburg, South Africa; d HIV, AIDS and STIs Cluster, National Department of Health, Pretoria, South Africa

**Keywords:** Pre-exposure prophylaxis, HIV prevention, condom use, young people, South Africa

## Abstract

**Background:** Pre-exposure prophylaxis (PrEP) for HIV prevention is safe and effective in reducing HIV incidence. However, more evidence of PrEP knowledge, willingness and distribution preferences is required for scale-up among young people at-risk.

**Objective:** To understand young people PrEP awareness, willingness and roll-out preferences.

**Methods**: Young people (18–24y) were selected through multi-stage sampling in a cross-sectional household survey in low-income communities. Self-administered interviews collected participants’ data about PrEP awareness, attitudes, willingness and HIV-risk practices. Data were descriptively analysed by gender. Regression models assessed factors associated with PrEP awareness and willingness by district.

**Results:** Of the 1917 participants interviewed 44.6% (men = 39.4% vs women = 49%, p = 0.001) were PrEP aware, 49.0% were willing to use PrEP. Participants most preferred PrEP distribution channels were public clinics (51.2%) and hospitals (23.8%). More men than women preferred distribution through schools (11.9% vs7.8%; p = 0.002) and NGOs (8.5%vs5.4%; p = 0.008). The biggest barrier to PrEP willingness was inadequate PrEP knowledge (10.0%) but more men than women disliked taking pills daily (4.1%vs2.0%; p-value = 0.007). Gendered determinants to use PrEP were side effects (51%; men = 47% vs women = 55%; p = 0.001) and pill effectiveness (29.5%; men = 26.4% vs women = 32.6%; p = 0.003). In both districts PrEP knowledge was associated with being female and media use. The associations between PrEP awareness and having multiple sexual partnerships, HIV knowledge, HIV self-test willingness and belonging to social clubs differed by district. PrEP willingness was positively associated with having TB and PrEP knowledge in each district but district differences were observed in media and occupation factors.

**Conclusions:** The study shows young people’s low levels of PrEP awareness. It also shows relatively increased willingness, gendered PrEP awareness and distribution preferences. Promoting youth’s PrEP awareness requires a multifarious media strategy.

**Abbreviations:** HIV: human immunodeficiency virus; AIDS: Acquired immunodeficiency syndrome; aOR: Adjusted Odds ratio; PLWH: People living with HIV; PrEP: Pre-exposure Prophylaxis; UNAIDS: Joint United Nations Programme on HIV and AIDS; uOR: Unadjusted odds ratio; TB: Tuberculosis; WHO: World health Organisation; MSM: Men who have sex with men

## Background

With 1.7 million [1.6 million–2.3 million] new HIV infections globally in 2018, having registered small declines since 2010 (2.1 million [1.6 million–2.7 million] [1], the world still has an unimaginable burden of HIV pandemic. New infections are far higher than the projected target of 500,000 per annum. Although the global picture is bleak, particularly in Sub-Saharan Africa which has two-thirds of all (38 million) HIV cases, the UNAIDS 2019 report suggests that gains in reducing HIV deaths and curtailing new infections in Eastern and Southern Africa were driving global progress around 2018 [1]. Such progress not only needs to be sustained but also to be accelerated through the use of new technologies and combination prevention methodologies. South Africa’s 7.9 million people living with HIV (14.0% adults) constitute the world’s largest national HIV burden [2,3]. South African young women (20–24 years) have a disproportionately high prevalence of up to 15.6% compared to their male counterparts with as low as 4.8%. HIV incidence among young people is 1.0% but is three times higher in women than in men (1.51% vs. 0.49%) [2]. This situation requires further interventions to curb new infections and deaths. The use of pre-exposure prophylaxis (PrEP) for HIV prevention is a new HIV prevention tool recently introduced for use in combination HIV prevention methods [4–9]. The HIV burden of young people in South Africa is influenced and compounded by many factors including early (<15y) sexual debut reported by young people aged 15–24 years which has risen from 8.5% in 2008 to 13.6% in 2017. Females aged 15–19 years having age-disparate relationships (partners who are 5+ more years older) which increased from 27.6% in 2008 to 35.8% is another important factor fuelling HIV burden. The HIV burden is also driven by multiple sexual partnerships in 25.5% males aged 15–24 years compared to women’s 5.1% and low condom use at last sex of 67.7% in males and 49.8% females aged 15–24 years [3]. These factors pose a great risk of HIV infection in young people in South Africa. The use of PrEP may meaningfully alter the trajectory of new infections driven by an array of these and other factors.

A global review of 561 PrEP studies showed that PrEP use for HIV prevention is an emerging but fast-growing field of study [10]. The review found that most studies were done cross-sectionally and focussed mainly on men who have sex with men (MSM). Only 3% of the studies were conducted among the younger population. The strong focus on MSM was associated with the 2012 WHO recommendation for the MSM to use PrEP to prevent HIV. However, since the recommendation to use PrEP was extended to include people at substantial risk, fewer studies were conducted to understand awareness, perceptions and uptake among other populations at substantial risk, including young women and men. As MSM drive the pandemic in settings such as the USA [11–13], so are young men and women driving the pandemic in Sub-Saharan Africa including South Africa [14,15]. It is therefore imperative to focus PrEP for HIV prevention research on this population in South Africa.

The earliest studies on PrEP for HIV prevention use were around 2006 and were mainly pilot and demonstration studies [16]. Through 2012 several key studies were published [10] giving enough and strong evidence to the WHO to recommend PrEP use among people at substantial risk [17] as initial guidelines were specific on MSM. Since the safety and effectiveness of PrEP have been established with no substantial changes in risk-taking sexual behaviours as previously assumed [18] and that it has been found to be cost-effective in modeling studies [19], implementation research should therefore follow to guide programming of PrEP use. This also requires understanding potential clients’ acceptability, knowledge and uptake including how the drug can be distributed in each setting. This is important because issues such as stigma and lack of awareness have been reported as barriers to PrEP uptake but in only a few settings studied [20,21]. More user dynamics, therefore, need to be understood. It is against this background that studying client-related aspects of PrEP awareness and uptake willingness is important in South African low-income communities.

By June 2018, 40 countries globally had some type of policy on oral PrEP use and these included 21 low- and middle-income countries [16]. In 2016 South Africa adopted a policy on PrEP. Policymakers, health workers and NGOs in sub-Saharan Africa also pledged willingness to support PrEP roll out [22]. However, there is still debate in South Africa among policymakers as to how it can be implemented in adolescents and young people. Also, in order to effectively implement PrEP, we must understand factors that correlate with a desire to use PrEP [23].

We, therefore, conducted a study to assess young people’s awareness, attitudes, and willingness to use PrEP as an HIV prevention tool. The study was part of a larger baseline assessment of young people’s knowledge attitudes and practices of HIV and TB in an ongoing HIV prevention programme [24]. The analysis in this paper is consequently aimed at providing insights on gendered aspects of PrEP among young people in South Africa.

## Methods

### Design and setting

The data for this paper come from a cross-sectional baseline survey conducted as part of a community-based HIV prevention interventional study on young men and women aged between 18 and 24 years. The intervention aimed to increase HIV knowledge, reduce risky sexual practices and increasing HIV testing and treatment in South African low-income communities in Nkangala district in Mpumalanga Province and OR Tambo district in the Eastern Cape Provinces. Although the districts’ areas where the study was conducted were both described as resource-limited, OR Tambo experienced higher levels of poverty compared Nkangala. Both districts had more than 98% Black Africans, 15.7% of the people had no formal schooling in OR tambo compared to 13.8% in Nkangala. Data were collected through a household survey. The detailed study methods were previously reported elsewhere [25].

### Sampling

We calculated a minimum sample size of 1816 participants for the study. To achieve this sample, population clusters in the districts were randomly sampled using the catchment areas of the public health facilities as the sampling frames. In each of the 22 selected clusters in both districts, households’ numbers based on the local government list of housing were followed serially and participants were enrolled consecutively, from one house to another. Only one participant who matched the inclusion criteria (aged 18–24 years, living in the selected district) per household was selected for interview. Where there were more than one eligible household members we selected the one whose first name appears first in alphabetical order. Potential participants were visited for interviews, if they were not available, they were visited three times before they were dropped from the survey.

### Questionnaire development

A questionnaire was designed to measure several variables regarding PrEP such as awareness, uptake willingness, preferred distribution channels and preferred information channels. To assess PrEP awareness, participants were asked if they ever heard of PrEP using the following question, ‘Have you ever heard about pre-exposure prophylaxis, which is also called PrEP?’ This was followed with a second question, ‘Have you ever heard about HIV medication that is taken to help prevent becoming infected with HIV?’. The two questions were meant to first assess if they just heard about it, and secondly to give them a clue of what PrEP was so that they can answer from an informed position. Answering yes to either or both questions were regarded as being aware of PrEP.

Participants’ knowledge of HIV was assessed using five questions following the UNAIDS conceptualisation of HIV knowledge [26] and previous South African national HIV surveys [27]. TB knowledge was assessed using eight questions that covered transmission, treatment and HIV/co-infection. Answering at least six questions was judged as knowledgeable of TB [25]. Four questions were used to judge a participant’s TB stigma through asking community attitudes towards people living with TB. Participants were also asked how frequently they used each of the following media sources for information – television, radio, social networking sites and WhatsApp platform. Each question was categorised as Never, once in a while/rarely, once a week, 2–6 days a week or every day of the week. Once in a while/rarely and once a week were regarded as occasional use while 2–6 days a week or everyday were categorised as frequent use of the media source. Willingness to HIV self-test was assessed using a question that asked if a participant was willing to self-test if it was given to them. An affirmative response was regarded as willing to self-test. Participants were asked if they used a condom at last sex to measure condom use at last sex. Participants were asked if they ever engaged in transactional sex. We also asked participants’ use of the media including social networking sites. Participant’s demographic characteristics were added to be able to describe their gender, age, occupation, education, socio-economic characteristics (possession of household goods, income source). The English questionnaire was also translated to and administered in participant’s first languages – isiNdebele and isiXhosa. The questionnaire was set into a RedCap electronic system for data collection [28,29].

### Data collection

Data collection procedures, ethics and instruments were pretested in the communities resulting in only minor corrections done to the questionnaire, translations and logistical issues. Male and female fieldworkers were engaged and received a seven-day training on recruitment, interviews, ethics, documentation and quality assurance. Fieldworkers were deployed and they recruited, enrolled and collected data from participants in the communities. Fieldworkers moved from house to house, being guided by aerial residential maps developed following the study sampling plan. After completing the relevant ethical, recruitment and enrolment process the fieldworker gave a participant a tablet with the questionnaire to self-complete the interview while the fieldworker was within reach to assist when prompted for help in navigating through the sections. Daily, weekly and monthly debriefings, monitoring and implementation meetings including tallying of interview participants by gender, cluster and district were done to ensure quality and completeness of data within the sampling confines. Data collection took place between October 2017 until January 2018.

### Data analysis

Data were analysed in Stata 13.0 [30]. We used descriptive analyses for participants’ demographic characteristics, PrEP awareness, HIV knowledge and risk, PrEP distribution, information needs, willingness and preferences as well as condom use at last sex. We conducted two multiple regression analyses. The first model assessed factors associated with PrEP awareness and the second model assessed factors associated with having willingness to use PrEP. For each model, we selected factors for inclusion using two methods. Firstly, we were guided by our knowledge of PrEP and possible associated factors based on the literature [23,31–33]. Secondly, we tested each variable through bivariate analysis using the threshold for variable selection for logistic regression described by Hosmer and Lemeshow [34]. Following this guidance, we, therefore, selected variables with a p-value of <.250 and added them into the respective stepwise multiple regression model. We controlled for participant’s demographic characteristics such as socioeconomic status and gender in the models. The model outputs are presented as adjusted Odd Ratios (aORs) in the results section.

### Ethics

The study received Ethics approval from the Foundation for Professional Development Research Ethics Committee. All participants interviewed provided written informed consent.

## Results

### Characteristics of the sample by PrEP awareness

A total of 1955 participants participated in the study. Table 1 shows characteristics of the sample by PrEP awareness. More than half the participants were still in school and were between 18 and 21 years old. Half were women and lived in OR Tambo district. Less than half (44.6%) the participants had awareness of PrEP. More TB knowledgeable participants reported being aware of PrEP than those who did not know TB (p = 0.027) but fewer HIV-knowledgeable participants reported PrEP awareness than those who were not HIV knowledgeable (p = 0.011). More PrEP-aware participants reported belonging to a social club grouping than those who did not belong to a social grouping (p = 0.010).

**Table 1.** Characteristics of the sample by PrEP awareness

	Total		PrEP awareness	No PrEP awareness	
	n/N	%	%	%	p-value
Gender: Female (vs male)	940/1843	51.0	49.0	51.0	**<0.0001**
Age: 18–21 (vs 22–24)	865/1823	47.5	47.3	47.5	0.935
In-school (vs out of school)	1018/1843	55.2	54.7	55.6	0.694
Social club member	756/1816	41.6	45.0	39.0	**0.010**
Social grant recipient	302/1829	16.5	17.3	15.9	0.428
Living in OR Tambo	906/1853	48.9	44.9	52.1	**0.002**
Have HIV knowledge	803/1785	45.0	41.7	47.7	**0.011**
Have TB Knowledge	1233/1708	72.2	74.9	70.0	**0.027**
Condom use at last sex	1111/1516	73.3	73.8	72.9	0.686

Table 2 shows PrEP awareness, attitudes and practices by gender. PrEP awareness differed by gender with more women (49.0%) than men (39.4%) reporting being aware of PrEP (p = 0.001). Half the participants (49.0) expressed willingness to use PrEP. See Table 2.

**Table 2.** PrEP awareness, attitudes and practices by gender (N = 1955)

	Total n/N	%	Male %	Female %	p-value
PrEP awareness	822/1853	44.4	39.4	49.0	**<0.001**
PrEP willingness	899/1834	49.0	48.1	50.0	0.418
**PrEP distribution channels**					
Private doctor	1476/1917	23.0	21.9	24.1	0.246
Hospital	457/1917	23.8	22.1	25.6	0.074
Clinic	936/1917	51.2	49.4	52.9	0.127
School	189/1917	9.9	11.9	7.8	**0.002**
Community health workers	228/1917	11.9	12.2	11.6	0.733
Non-governmental organisation (NGO)	133/1917	6.9	8.5	5.4	**0.008**
Traditional healer	20/1917	1.0	1.1	1.0	0.987
Mobile clinic	137/1917	7.2	8.0	6.3	0.169
Family member	38/1917	2.0	2.5	1.5	0.097
Other	16/1917	0.8	1.1	0.6	0.308
**Reasons for not using PrEP**					
Possible side effects	132/333	39.6	41.3	37.7	0.494
No adequate knowledge	189/333	56.8	53.1	61.0	0.143
Does not want family to know it	49/333	14.7	16.8	12.3	0.256
Fear to be more sexually active	47/333	14.1	13.4	14.9	0.690
Does not want a pill every day	58/333	17.4	21.8	12.3	**0.023**
Possibility of riskier life like not using a condom	36/333	10.8	11.7	9.7	0.560
**Things to know to decide to use PrEP**					
Side effects	980/1917	51.1	47.2	55.0	**0.001**
Place to get the pill	594/1917	31.0	30.4	31.6	0.559
Person who gives the medicine	323/1917	16.9	17.3	16.4	0.618
Duration of taking the pill	590/1917	30.8	29.2	32.3	0.140
How well the pill works	566/1917	29.5	26.4	32.6	**0.003**
How often the pill is to be taken	316/1917	16.5	14.7	18.3	**0.032**
Cost of the pill	461/1917	24.1	23.8	24.3	0.776
How the pill is taken	360/1917	18.8	17.7	19.9	0.226
**Young people’s preferred information channels about PrEP**					
Newspapers	658/1917	34.3	33.1	35.6	0.256
Billboard adverts	449/1917	23.4	24.6	22.3	0.222
School visits	967/1917	50.4	49.7	51.1	0.538
Television (TV) adverts	1123/1917	58.6	60.8	56.3	**0.046**
Brochures at health facilities	525/1917	27.4	26.4	28.4	0.328
Social media e.g. Facebook, WhatsApp	909/1917	47.4	45.5	49.4	0.085

### PrEP distribution channels

Participants were asked about PrEP distribution channels that they would consider receiving PrEP through. Table 2 shows the distribution channels by gender. More than half the participants (51.2%) preferred PrEP to be delivered through the public clinic. This was followed by participants who preferred the public hospital (23.8%) and private doctor (23%) and through community health workers (11.9%). Men and women differed in their distribution method preference in only two of the nine choices given – schools and non-governmental organisations (NGOs). More men (11.9%) than women (7.8%) preferred PrEP to be distributed to them through the school institution (p = 0.002) while more men (8.5%) than women (5.4%) preferred PrEP distribution through community NGOs (p = 0.008).

Slightly over half the participants (51.0%; 935/1834) expressed no willingness to use PrEP. Table 2 shows the reasons for not opting to use PrEP. The most common reason was not having adequate knowledge of PrEP (56.8%). This was followed by fear of the possible side effects of PrEP (36.6%). Other reasons mentioned include not wanting to take up a pill every day (17.4%), not wanting the family to know that one was taking PrEP (14.7%), fear to be more sexually active after initiating PrEP (14.7%), and the possibility of not using a condom while taking PrEP (10.8%). More men reported not wanting to take a pill daily (21.8%) than women (12.3%) (p = 0.023).

Participants were also asked about the things that they felt they needed to know before deciding to take PrEP. More than half (51.1%) the participants were concerned about the side effects of the PrEP pills. This was followed by almost a third who were concerned about a place where they could get a pill (31.0%), duration of taking the pill (30.8) and how well the pill works (29.5%). Gender differences were also observed: more women than men were worried about how the pill works (p = 0.003) and how often the pill is taken (p = 0.032). More women (55.0%) than men (51.1%) were concerned about the side effects (p = 0.001).

Participants were also asked about their preferred information channels to know more about PrEP. The most common medium of communication preferred was television adverts (58.6%) followed by school visits (50.4%) and the social media (47.4%). More women (56.3%) than men (60.8%) preferred the television adverts (p = 0.046).

### Factors associated with having PrEP awareness by district

There were differences and similarities in factors associated with PrEP awareness by district (see Table 3). In both districts being a woman and frequently listening to the radio were positively associated with PrEP awareness. In Nkangala district the use of the television was negatively associated with being PrEP aware while WhatsApp was positively associated with PrEP awareness. In OR Tambo occasional use of the radio, and social networking platforms were associated with having PrEP knowledge. Participants reporting willingness to self-test for HIV were less likely to have PrEP knowledge. In OR Tambo having at least three sexual partners in life, having HIV knowledge and belonging to community social clubs were associated with PrEP knowledge.

**Table 3.** Factors associated with having PrEP awareness

	Nkangala		OR Tambo	
Factors	aOR	95% CI	aOR	95% CI
Gender: Female	1.56	1.08–2.26	1.75	1.22–2.51
Watching television frequently (vs never)	0.52	0.31–0.88	NS	
Listening to the radio frequently (vs never)	3.1	1.73–5.57	2.13	1.48–3.05
Listening to the radio occasionally (vs never)	NS		2.18	1.43–3.33
Using social networking platforms	NS		2.28	1.22–4.27
Using WhatsApp frequently	1.62	1.21–2.16	NS	
Multiple sexual partners (3+)	NS		1.62	1.10–2.37
Willingness to self-test for HIV	0.66	0.50–0.88		
Having HIV knowledge	NS		0.65	0.45–0.94
Social club membership	NS		1.63	1.13–2.35

### *Factors associated with willingness to use PrEP by district* (Table 4)

We observed both similarities and differences in associations with willingness to use PrEP for HIV prevention by district of participants. In both districts having TB knowledge and PrEP awareness were strongly associated with reporting willingness to use PrEP. In Nkangala having accepting attitudes towards people living with HIV and being a student were positively associated with reporting willingness to use PrEP. The use of WhatsApp messaging service was positively associated with PrEP willingness while reporting unwillingness to self-test for HIV was associated with decreased chances of having willingness to use PrEP.

**Table 4.** Factors associated with willingness to use PrEP

	Nkangala		OR Tambo	
Factors	aOR	95% CI	aOR	95% CI
Having TB knowledge	1.89	1.31–2.72	2.73	1.67–4.49
Having PrEP awareness	1.83	1.29–2.60	2.95	2.00–4.36
Accepting attitudes towards people with TB	1.76	1.18–2.62	NS	
Listening to the radio frequently (vs never)	0.49	0.26–0.91	NS	
Being a student	1.45	1.02–2.06	NS	
Use of WhatsApp frequently	NS		1.80	1.14–2.84
Unwilling to self-test for HIV	NS		0.61	0.45–0.84

## Discussion

We found less than half the participants aware of PrEP. However, almost half were willing to use PrEP. The study also demonstrated the gendered nature of PrEP awareness as more women had knowledge of PrEP than men. In addition, the study found the importance of social media, HIV/TB knowledge and risk as the key influences of PrEP knowledge. We also found that the traditional media (radio and television) and modern social networking media services to be differentially associated with PrEP in each district. Not one media source proved to be consistent in educating and creating demand for PrEP and therefore a combination is needed. We, therefore, conclude that a robust multi-facetted media campaign, tailor-made for youth, be implemented to educate young people about PrEP and encouraging PrEP uptake to those at substantial risk. We also found TB and HIV factors to be important factors in PrEP awareness and use willingness. The knowledge of TB and PrEP has a positive bearing on the choice to take PrEP among young people. These findings should be interpreted in the context of WHO and South African policy for PrEP as an HIV prevention tool as shall be discussed in the following paragraphs.

While many studies on PrEP have focussed on high HIV-risk populations such as men who have sex with men, transgender people, sex workers, discordant couples [35–40] focus on the gendered differences of awareness of PrEP in the young men and women have been neglected. Our study provides an important finding on gender differences in PrEP awareness and uptake. Gender differences in PrEP knowledge follow society’s gender differences and access to community and health facilities. We found that while more men than women preferred schools and NGOs as distribution channels, more women than men preferred the health facilities as the PrEP delivery institution. Women have more access to health facilities than men and this happens through antenatal and postnatal care visits and therefore mentioned these health facilities for PrEP distribution. Contrastingly, men seemed to suggest those institutions that they have access to in the community as their preferred distribution channels and these were the NGOs and schools. Our results, therefore, suggest a differentiated nature of PrEP delivery channels among genders.

A sub-analysis of the participants who reported not willing to consider PrEP showed gendered reasons for refusal to use PrEP. Men were more likely than women to refuse to take up PrEP because they did not want to take a pill daily. Put differently, men had challenges in adhering to PrEP daily pill use. Challenges of adherence to ARV drugs have been reported more in men than in women in many studies globally [41–43]. Although family planning is different from HIV infection, they both share a number of things in common that are important for PrEP uptake [44]. For example, the fact that women take contraceptive pills daily can be used to influence them taking PrEP pills daily during the period they are at risk. Also that women can drop a method during periods that they are highly likely not having sexual relations can help us to understand that women can successfully take PrEP during the periods that they are at risk of HIV infection. These similarities between PrEP and family planning may help us to explain differences between men and women in PrEP willingness – as women find it easier to implement PrEP due to their experiences in using family planning methods. This is supported by current evidence of relatively high coverage of contraception use in South Africa which is half of all women of reproductive age while 9 in 10 have knowledge of oral contraception [45]. Hence, women did not seem to be bothered by regular PrEP pill taking when compared to men. Men would therefore need more education on adherence techniques to prepare them for optimum PrEP adherence.

Strong relationships were found between PrEP awareness and the media use in four media types (television, radio, WhatsApp, online social networks) tested in our models. Results show the radio, a traditional media communication source, to be a positive influencer in making people aware of PrEP in both districts. We also observed that among the modern social media sources, the WhatsApp (Nkangala district) and social networks (OR Tambo district) were common. That being a member of a social club was associated with PrEP awareness testifies to community integration. It shows that the youth are not isolated but forge networks and participate in them leading to gaining more awareness of issues around them. It also demonstrates the positive use of networks in their lives. Our results, therefore, support the notion that access to and participation in social networks is associated with better health outcomes [46,47], which is PrEP awareness in our study. The results show that a more differentiated media campaign will need to be strengthened and sustained for continued PrEP awareness. As the study recruited participants in both rural and urban communities our results show that not one media type will be adequate or preferred but a multiplicity of media types in an integrated manner for educational purposes. We make a few arguments to explain these media relationships with PrEP awareness. Since PrEP is a newer HIV prevention tool in the HIV prevention toolbox [48], information seems to spread faster and wider through the social networking sites and mobile-based platforms such as the WhatsApp [49,[50]. Young people seem to be interacting more on these platforms, hence gaining more awareness and knowledge. In addition, young people seem to have moved from acquiring new knowledge through the television due to the wider availability of social networking sites and WhatsApp which appear to be available among young people. However, some argue that data costs for social networking platforms and WhatsApp are still unaffordable to the South African masses who experience high unemployment and poverty. We recommend that huge investments be done in increasing PrEP awareness through the social networking sites most accessed by the youth. We also suggest that qualitative research be conducted to understand the reasons why negative associations were observed between PrEP awareness and the television use.

Having multiple sexual partners is a recognised HIV-risk factor in HIV-burdened countries of sub-Saharan Africa [15,51,52]. Our study found that multiple sexual partnerships were positively associated with knowing PrEP. It is commendable that people engaging in multiple sexual relationships know PrEP as an HIV prevention method because of their risky behaviour which requires HIV prevention. Being aware of PrEP is an opportunity towards preventing HIV through PrEP both for the public and for programme implementers to exploit for better outcomes. In addition, we found an association between willingness to self-test for HIV and being PrEP aware. Self-testing is a technique for people at risk to test themselves for HIV which in itself is a gateway to living positively [53]. That people who were willing to test themselves were aware of PrEP helps us to understand that people at risk have knowledge of HIV prevention methods and are willing to take precautionary measures to minimise risk using the newer and more personally controlled HIV prevention methods like PrEP.

Factors associated with willingness to use PrEP include having PrEP awareness, having TB knowledge and having accepting attitudes towards people living with TB. The association between TB factors and PrEP could be because participants with TB knowledge might have been exposed to chronic illnesses or its knowledge [25] which required them to prevent HIV which is a similar chronic condition. Since TB co-infects with HIV, some participants may have been exposed to HIV/TB information which then encouraged them to develop a willingness for PrEP. South Africa developed and implemented a programme of TB/HIV integration and management [54–56]. This programme recommends that people at risk of or actually testing positive to either TB or HIV be tested for the other infection. It is, therefore, possible for people with TB knowledge to be willing to enrol for PrEP. These assumptions for the relationship between TB and PrEP as mediated by HIV are important for the prevention of HIV through PrEP.

We also learnt from both districts that having PrEP awareness was associated with willingness to use PrEP. This may mean that in order to develop demand in PrEP uptake, programmes must first ensure that communities are aware of PrEP. Programmes for motivating young people to take up PrEP must first educate and make the communities aware of PrEP and its benefits and risks. Such education can follow the integrated media approach described above.

One of the limitations of the study is that it was cross-sectional in its design. Cross-sectional studies limit causal explanations due to the temporal nature of the relationships between the exposure and the outcome. For example, we were not able to establish if exposure to the media sources resulted in PrEP awareness or if non-condom use or multiple sexual partnerships made them aware or willing to use PrEP or vice versa. Also, our measurement of willingness was not based on offering PrEP to participants but only asking whether one wanted it if it was made available to them. Future studies can assess willingness with actually offering PrEP to participants. Nevertheless, we were able to learn from the study about young people’s awareness and willingness to use PrEP. Since HIV prevention remains critical among young people, this study’s findings will go a long way paving way for more intervention and implementation studies such as the distribution of PrEP medicines to young people including their preferred channels and factors that may hinder or favour PrEP implementation.

## Conclusions

The study found less than half the participants neither aware of PrEP nor willingness to use PrEP. It also found that not one media source proved to be consistent in educating and instilling demand for PrEP and therefore a combination is needed. It also found that knowledge of TB and PrEP have a positive bearing on the choice to take PrEP among young people. Willingness to use PrEP indicate opportunities for offering PrEP to young people in future. Our study is among the first to highlight young people’s PrEP uptake willingness at a population level in HIV-burdened communities. Our findings are important in outlining challenges and opportunities for PrEP rolled-out. In addition, our study focussed on young people, a population at greater risk for HIV infection in South Africa. This, therefore, calls for their prioritisation with HIV prevention interventions to reduce HIV infection.

## Data Availability

All data for this manuscript will be made available.

## Paper context

**What is already known?**

PrEP is safe and effective but awareness, willingness and distribution channels for young people need to be understood.

**What this paper adds**

Awareness among young people is low but uptake willingness is relatively high. Knowledge of PrEP is a barrier to uptake. Youth prefer PrEP to be dispensed through available public health system.

**What action needs to be taken?**

Media campaigns including addressing sexual risk factors are needed to increase PrEP awareness and uptake.
